# *Toona Sinensis* and *Moschus* Decoction Induced Cell Cycle Arrest in Human Cervical Carcinoma HeLa Cells

**DOI:** 10.1155/2014/121276

**Published:** 2014-01-08

**Authors:** Hong Zhen, Yifei Zhang, Zhijia Fang, Zhiwei Huang, Chongge You, Ping Shi

**Affiliations:** ^1^State Key Laboratory of Bioreactor Engineering, East China University of Science and Technology, 130 Meilong Road, Shanghai 200237, China; ^2^College of Chemistry, Chemical Engineering and Biotechnology, Donghua University, 2999 Renmin Road, Shanghai 201620, China; ^3^Lanzhou University Second Hospital, 82 Cuiying Gate, Lanzhou 730030, China

## Abstract

*Toona sinensis* and *Moschus* are two herb materials used in traditional Chinese medicine, most commonly for their various biological activities. In this study, we investigated the inhibitory effect of three decoctions from *Toona sinensis*, *Moschus,* and *Toona sinensis* and *Moschus* in combination on cell growth in several normal and cancer cell lines by cell viability assay. The results showed that the combined decoction exhibited the strongest anticancer effects, compared to two single decoctions. The observations indicated that the combined decoction did not induce cell apoptosis and autophagy in HeLa cells by fluorescence microscopy. Flow cytometry analysis revealed that the combined decoction arrested HeLa cell cycle progression in S-phase. After the decoction incubation, among 41 cell cycle related genes, eight were reduced, while five were increased in mRNA levels by real-time PCR assay. Western blotting showed that there were no apparent changes of protein levels of Cyclin E1, while P27 expression significantly declined and the levels of CDC7 and CDK7 obviously increased. The data suggest that the RB pathway is partially responsible for the decoction-induced S-phase cell cycle arrest in HeLa cells. Therefore, the combined decoction may have therapeutic potential as an anticancer formula for certain cancers.

## 1. Introduction

Traditional Chinese medicine (TCM) has been used in clinical practice for thousand years. Compound formula of TCM has been shown to exhibit synergism [1]. TCMs are used to restore overall healthful balance and normal body function in a holistic way due to their moderate treatment effects and lower side effects [2]. These features made themselves popular in China.

*Toona sinensis* is a type of arbor that is widely distributed in Asia. The leaves and young shoots are used as vegetable in China and Malaysia [3, 4]. In fact, it has long been used in TCM for a wide variety of conditions. The leaf extracts showed various biological activities, including anticancer [5–9], antidiabetes [10], and antioxidant [11] effects, as well as inhibiting Leydig cell steroidogenesis [12] and suppressing brain degeneration in senescence-accelerated mice [13]. The bark is used as astringent and depurative, the powdered root is used as a corrective, and the fruits are used as an astringent and for the treatment of eye infection [14, 15].

Musk, a ventral glandular secretion of the male musk deer, is also a precious and wide applied material in TCM [16–18]. As a major Chinese herbal material, musk was firstly recorded in Shen Nong Ben Cao Jing (The Herbal Classic of the Divine Plowman) in about 2700 BC and has been widely used for thousands of years. Now it is officially listed in Chinese Pharmacopoeia as *Moschus*, recognized as an important medicinal material with many pharmacological activities, including resuscitation, activating blood to promote menstruation, detumescence, and analgesia [19].

The* Toona sinensis* and *Moschus* decoction has been used as a folk medicine for patients with liver cancer to alleviation of the symptom in Gusu province of China, while single *Toona sinensis* or *Moschus* shows no effect on these patients. The probable mechanism is unclear. In the present study, we investigated the effects of *Toona sinensis *and *Moschus* decoction on cell growth inhibition in several normal and cancer cell lines and explored its underlying molecular mechanism in human cervical carcinoma HeLa cells. Our results indicate that *Toona sinensis *and *Moschus* decoction inhibits HeLa cell growth by inducing cell cycle arrest at S-phase via regulation of some cell cycle related proteins. The findings provide evidence that *Toona sinensis *and *Moschus* decoction may have potential in the therapeutic use for some cancers.

## 2. Materials and Methods

### 2.1. Cell Lines and Cell Culture

The mouse embryo fibroblast cell line NIH3T3 was obtained from ATCC (Manassas, VA) and human hepatoma cell line SMMC-7721, cervical carcinoma cell line HeLa, and liver cell line QSG-7701 were supplied by the Type Culture Collection of the Chinese Academy of Sciences (Shanghai, China). They were maintained in RPMI 1640 medium (HeLa, SMMC-7721 and QSG-7701) or Dulbecco's modified Eagle's medium (NIH3T3), supplemented with 10% fetal bovine serum (GIBCO, USA) and 1% penicillin-streptomycin (10,000 U/mL penicillin and 10 mg/mL streptomycin, Solarbio Science and Technology, Beijing, China) at 37°C in humidified air with 5% CO_2_.

### 2.2. Herbal Extract and Treatments

The raw herbs of *Toona sinensis* bark and *Moschus* were purchased from Gansu province of China. Both of them were identified by Professor Guichen Chen from Northwest Institute of Plateau Biology, Chinese Academy of Sciences. The bark of *Toona sinensis* was firstly washed to remove all contaminants and then cut into small pieces. The decoction was prepared by mixing *Toona sinensis* bark and *Moschus* 50 : 1 weight ratio. The mixture was allowed to soak with 10-fold of water for 2 h, followed by extraction at 100°C for 3 h. The water extract was then dried at 50–60°C and stored at room temperature. The similar amount of *Toona sinensis* bark and *Moschus* was extracted as controls according to the above procedure, respectively. For all experiments, the herbal preparation was weighed out and dissolved in culture medium to a concentration of 5 mg/mL and was immediately added at the indicated concentrations in cultured cells.

### 2.3. Cell Viability Assay

Cell viability was evaluated by Cell Counting Kit-8 (Dojindo, Tokyo, Japan) according to the manufacturer's instruction. Cells were plated in 96-well micro-well plates in 0.1 mL volumes of medium with 10% FBS, at a density of 1 × 10^5^ cells/well. Absorbance was measured at 450 nm with a iMark Microplate Absorbance Reader (Bio-Rad Laboratories Inc., Hercules, CA, USA).

### 2.4. Hoechst 33258 Staining

The cells at 2 × 10^5^ cells/mL were seeded in 24-well plates and treated with different concentrations of the decoctions for 48 h. They were fixed in 4% paraformaldehyde in 4°C refrigerator for 30 min and then stained with 5 *μ*g/mL Hoechst 33258, a DNA-specific fluorescent dye, for 10 min at 37°C. The stained cells were observed under a fluorescence microscope (Olympus BX51, Tokyo, Japan).

### 2.5. MDC Autophagy Staining

For autophagic fluorescence observation, cells were grown on coverslips and treated with the decoction. After 48 h, cells were stained with 0.05 mM MDC at 37°C for 1 h. The cellular fluorescence was observed using Nikon Eclipse Ti-S inverted fluorescence microscope (Tokyo, Japan) with 490 nm band-pass excitation filter and 515 nm long-pass barrier filter.

### 2.6. Cell Cycle Analysis

Cellular DNA content was determined by flow cytometry. After incubation with the decoction for 24 h, cells were harvested, washed twice with ice-cold PBS, and fixed with 70% ethanol overnight at −20°C. Cells were then washed twice with ice-cold PBS and resuspended in PBS. RNA was digested with RNase A (100 *μ*g/mL) and DNA was stained with PI (50 *μ*g/mL). After incubation for 30 min in the dark at 37°C, stained cells were analyzed by a FACScan flow cytometry and CellQuest analysis software (Becton Dickinson, San Jose, CA).

### 2.7. RNA Extraction and Real-Time PCR

The total RNA extraction was performed using TRNzol Reagent (Tiangen, Beijing, China) according to the manufacturer's instructions. Complimentary DNA (cDNA) was prepared from the total RNA using FastQuant RT kit (With gDNase) (Tiangen, Beijing, China).

The real-time PCR analysis was performed in triplicate assays using SuperReal PreMix Plus (SYBR Green) according to the manufacturer's instructions (Tiangen, Beijing, China) in a CFX96 Real-Time PCR Detection System (Bio-Rad, USA). The reactions were as follows: activation of the Taq DNA polymerase at 95°C for 15 min and 40 cycles of 95°C for 10 s and 60°C for 32 s. We quantified 41 genes based on a cell cycle-associated cDNA microarray (CTBioscience, Changzhou, China). The symbols of the 41 genes are listed in Table 1. *Gapdh, *β*-actin, B2M, HPRT1, and OAZ1 *were selected as internal controls. The relative changes in gene expression were calculated using the equation: 2^−ΔΔCT^, as Livak described [20].

### 2.8. Western Blot

After cells were lysed with ice-cold RIPA lysis buffer (Beyotime Institute of Biotechnology, Changzhou, China) for 30 min on ice, lysates were centrifuged at 13,000 ×g with 20 min and the supernatants were used as total cell lysates. Protein concentration was determined by Bradford protein assay (Bio-Rad, USA). A quantity of 50 *μ*g total protein per lane was separated by 12% sodium dodecyl sulfate-polyacrylamide gel electrophoresis (SDS-PAGE) and transferred to Amersham Hybond-P polyvinylidene fluoride (PVDF) membranes (GE Healthcare, Buckinghamshire, UK). Membranes were blocked with 5% milk powder in 0.05% Tween-TBS, incubated with the specific antibodies, including Cyclin E1, p27, and CDK7 and CDC7 rabbit monoclonal antibodies (Epitomics, Burlingame, CA, USA). Detection of the target proteins on the membranes was performed using the ECL western blotting detection reagents. Signal density was detected using the MF-ChemiBIS Family including gel capture software (DNR Bio-Image system, Ltd., Jerusalem, Israel) and quantitatively analyzed by densitometry using the Image J software.

### 2.9. Statistical Analysis

Values were expressed as means ± standard deviations. Statistical analysis was performed using Student's *t*-test. Values of *P* < 0.05 were considered to be statistically significant.

## 3. Results

### 3.1. The *Toona Sinensis* and *Moschus* Decoction Inhibits Cell Proliferation

**Figure 1:**
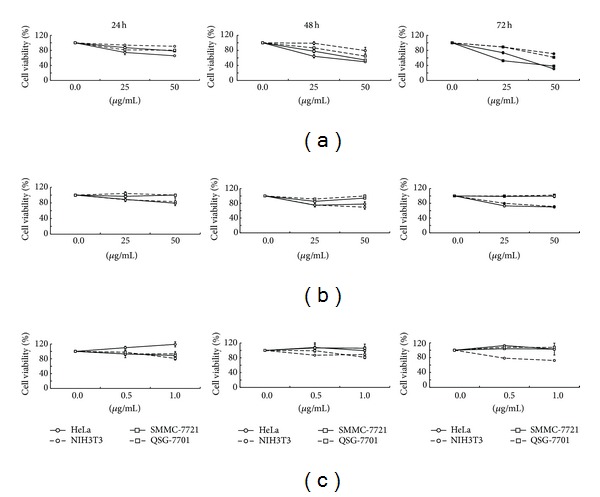
The effects of the three decoctions, including *Toona sinensis* and* Moschus* mixture, single *Toona sinensis* and *Moschus, *on cell viability in HeLa, SMMC-7721, NIH3T3, and QSG-7701 cells. (a) Cells were incubated without or with 25 and 50 *μ*g/mL of the mixture decoction and the effects on growth inhibition were in a concentration- and time-dependent manner. (b) Cells were incubated without or with 25 and 50 *μ*g/mL of the single *Toona sinensis* decoction. The percentage of viable cells was no less than 70% among all of the treated cells. (c) According to the weight ratio of the *Toona sinensis *and *Moschus* mixture decoction, the single *Moschus *concentration was set as 0.5 and 1 *μ*g/mL. In contrast to NIH3T3 and QSG-7701 cells, HeLa and SMMC-7721 cells showed no statistical decrease in the percentage of cell viability. The data are presented as the mean ± S.D. (for each group, *n* = 3).

Using the WST-8 based Colorimetric Assay Cell Counting Kit-8, the effect of the *Toona sinensis* and *Moschus* decoction on cell viability was measured. As shown in Figure 1(a), cells proliferation of human cervical carcinoma cell line HeLa and hepatoma cell line SMMC-7721 was inhibited by 50%, 70%, 46%, and 62% after 50 *μ*g/mL decoction treatments for 48 h and 72 h, respectively. Compared to the two carcinoma cells, mouse embryo fibroblast cell line NIH3T3 and normal liver cell line QSG-7701 showed that cell viability decreased by 29% and 38% at 50 *μ*g/mL decoction treatment for 72 h (Figure 1(a)). Obviously, the cell growth inhibitory effects of the decoction on the two cancer cells were stronger than those on the normal cells. Furthermore, the data indicates that the decoction inhibited the growth of cancer cells in a concentration- and time-dependent manner (Figure 1(a)).

At the same time, cells were treated by the single* Toona sinensis* or* Moschus* decoction. As shown in Figure 1(b), after the treatment of 50 *μ*g/mL single *Toona sinensis* decoction for 72 h, the percentages of cell viability were 70%, 71%, 99%, and 99.8% in HeLa, SMMC-7721, NIH3T3, and QSG-7701 cells, respectively. According to the weight ratio of the *Toona sinensis* and *Moschus* decoction, the single *Moschus *concentration was set as 0.5 *μ*g/mL and 1 *μ*g/mL. As shown in Figure 1(c), after the treatment of 1 *μ*g/mL single *Moschus* for 24 h, the percentages of cell viability were 122.8%, 88.8%, 93.3%, and 81.1% in HeLa, SMMC-7721, NIH3T3, and QSG-7701 cells, respectively. In contrast to NIH3T3 and QSG-7701 cells, HeLa and SMMC-7721 cells showed no statistical decrease in the percentage of cell viability (Figure 1(c)). In order to further understand the cell growth inhibition of *Moschus*, we used 25 *μ*g/mL and 50 *μ*g/mL* Moschus* to treat the cells. The results were similar as those in low *Moschus *concentrations (data not shown). Of important note is that the *Toona sinensis* and *Moschus *decoction did induce significant decrease in the viability of human carcinoma cells, other than single* Toona sinensis* or *Moschus *decoction (Figure 1).

### 3.2. The *Toona Sinensis* and *Moschus* Decoction Does Not Induce Cell Apoptosis and Autophagy

**Figure 2:**
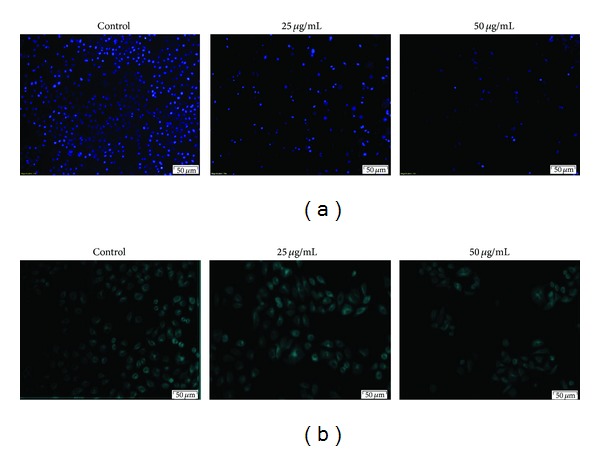
The morphologic changes of HeLa cells treated with the *Toona sinensis* and *Moschus* decoction for 24 h and then were observed by fluorescence microscopy after staining with Hoechst 33258 (a) and MDC dye (b). No apoptotic body and acidic vesicles were seen. The scale was 50 *μ*m at the bottom right corner in each picture.

We sought to explore a possible explanation for its inhibition of cancer cell growth. Hoechst 33258, a DNA-specific fluorescent dye, was chosen to observe the morphologic characteristics of apoptosis in HeLa cells. As shown in Figure 2(a), no apoptotic cells can be found after the decoction treatment for 48 h by fluorescence microscopy. Based on the above observations, which indicate that apoptosis is not the cause of cell death induced by the decoction, we next questioned if it induces autophagy, a second form of cell death, in HeLa cells. To address this issue, vital staining of HeLa cells treated with the decoction for 48 h was performed with MDC dye, a selective fluorescent marker of autophagic vesicles. The dye shows diffuse staining in nonautophagic cells but exhibits punctuate vesicular staining when autophagy is induced. As shown in Figure 2(b), no acidic vesicles were observed. Likewise, the decoction did not induce autography in HeLa cells.

### 3.3. The *Toona Sinensis* and *Moschus* Decoction Induces Cell Cycle Arrest at S-Phase

**Figure 3:**
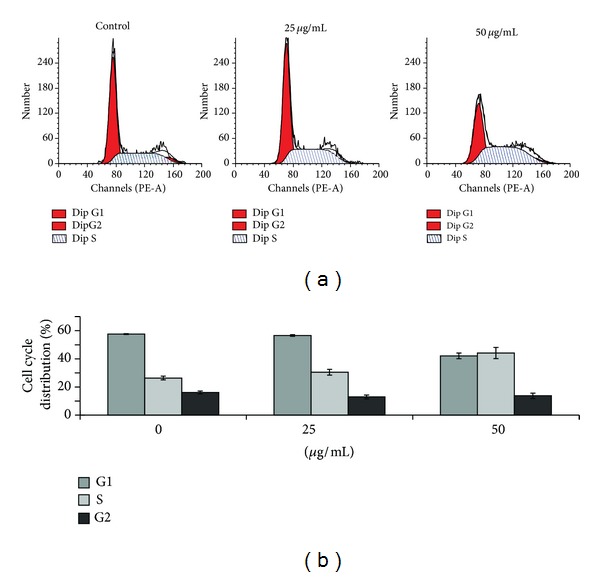
Effect of the *Toona sinensis *and *Moschus* decoction on the cell cycle distribution of HeLa cells. (a) Analysis of the various phases of the cell cycle by flow cytometry. A representative profile of cell cycle distribution in three independent experiments treated with 0, 25, and 50 *μ*g/mL for 24 h. (b) The percentage of cell cycle distribution after the decoction treatment. Data are presented as the mean ± S.D. of three independent experiments at each concentration.

To examine whether the *Toona sinensis* and *Moschus* decoction, induced growth inhibition was associated with cell cycle regulation, the cell cycle distribution was analyzed by flow cytometry. After HeLa cells were incubated with the decoction for 24 h, cells were harvested and further prepared for cell cycle analysis. Figures 3(a) and 3(b) showed that cells accumulated in the S phase of the cell cycle after the decoction treatment, whereas the percentage of cells in G_0_/G_1_ phase reduced significantly. Especially, the percentage of cells in S phase increased by 68% when the decoction concentration was 50 *μ*g/mL, compared to that of the untreated control (Figure 3).

### 3.4. Changes of Cell Cycle Related Genes mRNA and Proteins Expression

**Figure 4:**
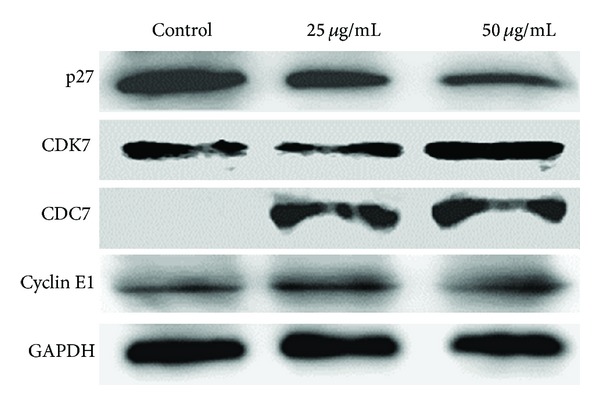
Effect of the *Toona sinensis *and *Moschus* decoction on the expression of p27, CDK7, CDC7, and Cyclin E1 in HeLa cells by western blotting. GAPDH was used as the loading control.

In order to determine whether the *Toona sinensis* and *Moschus* decoction was able to regulate cell cycle related genes expression, real-time PCR experiment was performed in the cell extracts prepared from HeLa cells. The results showed that the decoction incubation reduced the levels of *CCNA1, CCND1, CCND2, CCNH, CDKN1B, MCM2, ORC6L,* and *WEE1* and increased the levels of* CDC7, CDK6, CDK7, E2F3,* and *RB1* (Table 1). Taken together, among 41 detected genes, the mRNA levels of 13 genes were changed by the decoction. Next, western blotting with specific antibodies was done to detect the changes of related proteins expression levels. We found reduction of p27 and increase of CDK7 after the treatment of the decoction (Figure 4). The level of Cyclin E1 treated with the decoction showed no significant alteration as compared to the untreated control (Figure 4). In summary, the decoction regulates mRNA and subsequent protein levels of certain cell cycle-related genes to induce S-phase cell cycle arrest.

## 4. Discussion

Different from the orthodox medicine focusing on a specific pathogenic process [21], TCMs have been recognized as a popular complementary and alternative medicines, emphasizing on individualized diagnosis and treatment of patients; maximizing the body's inherent healing ability; treatment of the “whole” person by addressing their physical, mental, and spiritual attributes [22, 23]. Multiple agents contained in each formula, at least in some formulas, could hit multiple targets and exert synergistic therapeutic efficacies [24, 25]. In our study, it is consistent that the *Toona sinensis* and *Moschus* decoction shows stronger anticancer effects than either single *Toona sinensis* decoction or *Moschus* one by cell viability assay.

**Figure 5:**
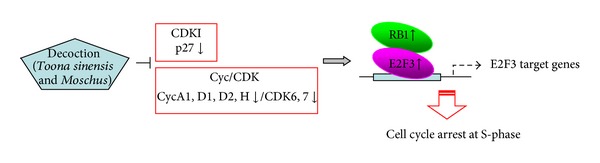
A scheme of the effect of the *Toona sinensis* and *Moschus* decoction on cell cycle arrest in HeLa cells. The RB pathway (CDK inhibitor-Cyc/CDK-RB-E2F3) is partially responsible for the decoctioninduced S-phase cell cycle arrest.

Our subsequent investigation was focused on the mechanisms of the *Toona sinensis* and *Moschus* decoctioninduced anticancer effects in HeLa cells. The major findings of this study were that the decoction induces cell cycle arrest in S-phase. Moreover, we showed that the cell-cycle arrest was accompanied by down-regulation of *CCNA1, CCND1, CCND2, CCNH, CDKN1B, MCM2, ORC6L,* and *WEE1 *and upregulation of* CDC7, CDK6, CDK7, E2F3,* and *RB1*. Western blot also confirmed the consistent changes of several corresponding proteins. It is well known that eukaryotic cell cycle is driven by an ordered activation of a family of protein kinases, cyclin-dependent kinases, which are composed of a catalytic subunit CDK and a positive regulatory subunit cyclin [26]. Each CDK/cyclin complex is considered to play specialized roles at a defined stage of cell cycle, through phosphorylation of its specific substrate proteins [27–30]. Our results demonstrated that there were no apparent changes of both the mRNA and protein levels of Cyclin E1 in the decoction treated cells. In contrast, P27 expression significantly declined and the level of CDK7 obviously increased in accordance to cell cycle arrest in the S-phase. All these results indicate that the effect of the *Toona sinensis* and *Moschus* decoction on cell cycle arrest may be a transcriptional event regulating the certain cell cycle related proteins. A schematic presentation of cell cycle arrest at S-phase in our study is shown in Figure 5. It seems that the RB pathway (CDK inhibitor-Cyc/CDK-RB-E2F3) is partially responsible for the decoctioninduced S-phase cell cycle arrest in HeLa cells.

In *Moschus*, many active compounds have been identified, such as muscone, stanolone, muscopyridine, androstane-3, 17-dione, hydroxymuscopyridine, and cholic acid. They are mainly classified as three major types including flavonoides, alkaloids, and steroids [31]. Hitherto, no reports show that these individual active ingredients exhibit effect on cell cycle progression. Regarding *Toona sinensis*, a number of compounds, including gallic acid, retinoid, vitamins B and C, o-coumaric acid, methyl gallate, ethyl gallate, kaempferol, kaempferol-3-O-*β*-D-glucoside, quercetin, quercitrin, quercetin-3-O-*β*-D-glucoside, and rutin, were identified [32, 33]. Among them, gallic acid has been shown to block the growth of DU145 cells at G2/M phases [34]. According to our results, the decoction induces S-phase cell cycle arrest in HeLa cells, which suggests that the cell cycle arrest is induced by compound(s) other than gallic acid. It is known that the use of complex medicinal formulation exhibits synergism of the multiple ingredients or drug-drug interactions [1]. The effect may not be achieved by individual active ingredients. Therefore, future studies in identifying the complex interactions of different chemical components of the *Toona sinensis* and *Moschus* will be helpful to evaluate the anticancer effect of the decoction.

## 5. Conclusion

This study is the first to report the anticancer activity of decoction of *Toona sinensis* and *Moschus* in combination. Furthermore, we have shown that the *Toona sinensis *and* Moschus* decoction exhibits its anticancer effects by inducing cell cycle arrest in S-phase via mRNA regulation of cell cycle related proteins in HeLa cells. Our data provide strong evidence that the *Toona sinensis* and *Moschus* decoction could be potentially utilized for the treatment of some cancers.

## Figures and Tables

**Table 1:** Effect of the *Toona sinensis* and *Moschus* decoction on the mRNA expression of 41 cell cycle related genes. HeLa cells were treated with 50 *µ*g/mL of the decoction for 24 h. The data are presented as the mean ± S.D. (for each group, *n* = 3).

No.	Symbol	Gene name	Accession no.	Fold change
1	CCNA1	Cyclin A1	NM_003914	0.47*
2	CCNA2	Cyclin A2	NM_001237	1.76
3	CCNB1	Cyclin B1	NM_031966	1.79
4	CCND1	Cyclin D1	NM_053056	0.26*
5	CCND2	Cyclin D2	NM_001759	0.02*
6	CCND3	Cyclin D3	NM_001760	0.96
7	CCNE1	Cyclin E1	NM_057182	0.51
8	CCNE2	Cyclin E2	NM_057749	1.07
9	CCNH	Cyclin H	NM_001239	0.47*
10	CDC25A	Cell division cycle 25 homolog A (*S. pombe*)	NM_201567	1.64
11	CDC6	Cell division cycle 6 homolog A (*S. cerevisiae*)	NM_001254	0.91
12	CDC7	Cell division cycle 7 homolog A (*S. cerevisiae*)	NM_003503	4.09**
13	CDK2	Cyclin-dependent kinase 2	NM_052827	1.01
14	CDK4	Cyclin-dependent kinase 4	NM_000075	1.02
15	CDK6	Cyclin-dependent kinase 6	NM_001259	2.53**
16	CDK7	Cyclin-dependent kinase 7	NM_001799	13.37**
17	CDKN1A	Cyclin-dependent kinase inhibitor 1A (p21, Cip1)	NM_078467	1.15
18	CDKN1B	Cyclin-dependent kinase inhibitor 1B (p27, Kip1)	NM_004064	0.47*
19	CDKN2A	Cyclin-dependent kinase inhibitor 2A	NM_058197	0.88
20	CDKN2B	Cyclin-dependent kinase inhibitor 2B (p15, inhibits CDK4)	NM_078487	0.88
21	CDKN2C	Cyclin-dependent kinase inhibitor 2C (p18, inhibits CDK5)	NM_078626	0.76
22	E2F1	E2F transcription factor 1	NM_005225	0.96
23	E2F2	E2F transcription factor 2	NM_004091	1.17
24	E2F3	E2F transcription factor 3	NM_001949	2.25**
25	HDAC1	Histone deacetylase 1	NM_004964	0.70
26	MCM2	Minichromosome maintenance component 2	NM_004526	0.47*
27	MCM3	Minichromosome maintenance component 3	NM_002388	0.87
28	MCM4	Minichromosome maintenance component 4	NM_182746	1.13
29	MCM5	Minichromosome maintenance component 5	NM_006739	0.92
30	MCM6	Minichromosome maintenance component 6	NM_005915	0.85
31	MCM7	Minichromosome maintenance component 7	NM_182776	1.31
32	ORC1L	Origin recognition complex, subunit 1-like (yeast)	NM_004153	0.94
33	ORC2L	Origin recognition complex, subunit 2-like (yeast)	NM_006190	1.24
34	ORC6L	Origin recognition complex, subunit 6-like (yeast)	NM_014321	0.42*
35	PCNA	Proliferation cell nuclear antigen	NM_182649	0.87
36	PKMYT1	Protein kinase, membrane associated tyrosine/threonine 1	NM_182687	1.13
37	RB1	Retinoblastoma 1	NM_000321	2.30**
38	RBL1	Retinoblastoma-like 1 (p107)	NM_183404	1.18
39	SKP2	S-Phase kinase-associated protein 2, E3 ubiquitin protein ligase	NM_032637	1.67
40	TFDP1	Transcription factor Dp-1	NM_007111	1.28
41	WEE1	WEE 1 homolog (*S. pombe*)	NM_003390	0.47*
42^#^	B2M	Beta-2-microglobulin	NM_004048	
43^#^	ACTB	Beta actin	NM_001101	
44^#^	GAPDH	Glyceraldehyde-3-phosphate dehydrogenase	NM_002046	
45^#^	HPRT1	Hypoxanthine phosphoribosyltransferase 1	NM_000194	
46^#^	OAZ1	Ornithine decarboxylase antizyme 1	NM_004152	

^#^The B2M, ACTB, GAPDH, HPRT1, and OAZ1 were selected as internal controls.

*Ratio < 0.5. **Ratio > 2.

**Means that “ratio > 2”.
